# Differential Kinase Activation in Peripheral Blood Mononuclear Cells from Non-Small-Cell Lung Cancer Patients Treated with Nivolumab

**DOI:** 10.3390/cancers11060762

**Published:** 2019-05-31

**Authors:** Gaëlle Noé, Audrey Bellesoeur, Lisa Golmard, Audrey Thomas-Schoemann, Pascaline Boudou-Rouquette, Manuela Tiako Meyo, Alicja Puszkiel, Jennifer Arrondeau, Jérome Alexandre, François Goldwasser, Benoit Blanchet, Michel Vidal

**Affiliations:** 1 Biologie du médicament-toxicologie, Hôpital Cochin, AP-HP, 75014 Paris, France; gaelle.noe@aphp.fr (G.N.); audrey.thomas@aphp.fr (A.T.-S.); alicjapuszkiel@gmail.com (A.P.); benoit.blanchet@aphp.fr (B.B.); 2 UMR8038 CNRS, U1268 INSERM, Faculty of Pharmacy, University Paris Descartes, PRES Sorbonne Paris Cité, 75006 Paris, France; audrey.bellesoeur@aphp.fr (A.B.); manuelatiako@gmail.com (M.T.M.); 3 Department of Medical Oncology, Hôpital Cochin, AP-HP, 75014 Paris, France; pascaline.boudou@aphp.fr (P.B.-R.); jennifer.arrondeau@aphp.fr (J.A.); jerome.alexandre@aphp.fr (J.A.); francois.goldwasser@aphp.fr (F.G.); 4 Biopathology Department, Institut Curie, 75005 Paris, France; lisa.golmard@curie.fr; 5 Immunomodulatory Therapies Multidisciplinary Study group (CERTIM), Hôpital Cochin, AP-HP, 75014 Paris, France; 6 U1016 INSERM, UMR 8104 CNRS, UMR-S1016, CARPEM, Université Paris Descartes, Sorbonne Paris Cité, 74014 Paris, France

**Keywords:** nivolumab, kinome, PBMC, non-small-cell lung cancer, immunotherapy

## Abstract

In the era of precision medicine, research of biomarkers for identification of responders to nivolumab therapy is a major challenge. Peripheral blood mononuclear cells (PBMC) could be an interesting surrogate tissue for identifying pharmacodynamic biomarkers. The aim of this exploratory study was to investigate the global serine/threonine kinase (STK) activity in PBMC from non-small-cell lung cancer (NSCLC) patients using a high throughput kinomic profiling method. PamChip^®^ microarrays were used to explore the STK kinomic profile in PBMC from 28 NSCLC patients before nivolumab initiation (D0) and on day 14 (D14) of the first administration. Two clusters of patients (A and B) were identified at D0, median overall survival (OS) tended to be longer in cluster A than in B (402 vs. 112.5 days, respectively; *p* = 0.15). Interestingly, the PD-L1 tumor cell score (*p* = 0.045), the count of CD8+ cells (*p* = 0.023) and the total body weight (*p* = 0.038) were statistically different between the clusters. On D14, clusters C and D were identified. Greater activity of most STK, especially those of the PI3K/Akt signaling pathway, was noticed among cluster C. No significant difference between C and D was observed regarding OS. Considering the small number of patients, results from this preliminary study are not conclusive. However, the 4-fold longer median OS in cluster A paves the way to further investigate, in a larger cohort of NSCLC patients, the benefit of basal STK kinomic profile in PBMC to identify responders to nivolumab therapy.

## 1. Introduction

Lung cancer is the leading cause of cancer death worldwide, with 1.825 million diagnoses and 1.59 million deaths in 2012 [1]. Non-small-cell lung cancer (NSCLC) accounts for 85% to 90% of all lung cancers. In the last years, the use of comprehensive mutation analysis technologies has accelerated the identification of genetic aberrations in NSCLC and therefore the clinical development of new targeted therapies such as EGFR and ALK inhibitors [2]. Additionally, a better understanding of the antitumor immunity highlighted the key role of immune checkpoints such as cytotoxic T lymphocyte antigen-4 (CTLA-4) and programmed death 1 (PD-1) in immune escape mechanisms [3]. The anti-PD-1 monoclonal antibody nivolumab binds to the PD-1 receptor and blocks its interaction with its ligands PD-L1 and PD-L2, thereby releasing PD-1 pathway-mediated inhibition of the immune response, including anti-tumor immune response [4]. Two phase III trials showed that nivolumab improves overall survival, response rate, and progression-free survival compared to docetaxel in patients with advanced, previously treated squamous-cell and nonsquamous NSCLC [5,6]. Nivolumab is currently approved for the treatment of patients with advanced-stage NSCLC following progression on or after platinum-based chemotherapy.

New challenges related to treatment optimization and economic issues are emerging with the introduction of PD-1/PD-L1 checkpoint inhibitors such as nivolumab [7,8]. Indeed, only 20% of NSCLC patients respond to nivolumab therapy and approximately two-thirds of these responses are durable. In the era of precision medicine, it is essential to identify the main factors of treatment failure in the other 80% of patients. The tumor expression of PD-L1 was initially suggested as a biomarker to predict response to anti-PD1/PD-L1 therapies. The Phase III trial conducted in nonsquamous NSCLC treated with nivolumab was in favor of a predictive value [6], while in the phase III trial in squamous NSCLC the expression of PD-L1 did not correlate with patients’ outcomes [5]. Recently, the Food and Drug Administration approved the tumor expression of PD-L1 as a companion test for PD-1/PD-L1 checkpoint inhibitors. However, clinical activity and durable response can be observed in some NSCLC patients in whom tumor expression of PD-L1 is negative. Taken together, these results suggest that the sensitivity of PD-L1 assay is not sufficient to discriminate nonresponders from responders, as confirmed in two phase III studies with first-line pembrolizumab [9] or nivolumab [10]. In this context, the research of reliable predictive biomarkers is always ongoing in order to identify NSCLC patients who will benefit the most from treatment with nivolumab.

The use of kinomic approach based on global kinases activities is currently spreading to seek prognostic and therapy-predictive biomarkers in oncology [11,12,13,14,15,16,17]. Two studies applying kinomic approach have been conducted in NSCLC patients. Hilhorst et al. showed that a classification based on basal kinase activity profiles in tumor tissue predicted erlotinib response in 14 NSCLC patients in a neoadjuvant setting [18]. The model was then validated in an external cohort of NSCLC patients (*n* = 13) [19]. Anderson et al. recently demonstrated the feasibility of using kinomic profiling of electromagnetic navigational bronchoscopy specimens for the research of future biomarkers in NSCLC patients [16]. As far as we know, no study has still investigated the kinomic profile of NSCLC patients treated with immunotherapy. Given that PD-1 is both largely expressed on lymphocytes and is the pharmacological target of nivolumab, it would be interesting to explore the kinomic profiles in peripheral blood mononuclear cells (PBMC) from NSCLC patients treated with nivolumab. This may provide helpful information to identify the pharmacological effect of nivolumab on signaling pathways in lymphocytes, to elucidate resistance mechanisms and to seek circulating biomarkers.

The aims of the present study were firstly to characterize (a) the global serine/threonine kinase (STK) activity in PBMC from NSCLC patients before nivolumab treatment; (b) the STK kinomic profile in PBMC just prior to the second infusion of nivolumab. A high throughput kinomic profiling method was employed for these purposes.

## 2. Results

### 2.1. Patients

Twenty-eight NSCLC patients treated with nivolumab were included. Clinical and biological baseline characteristics are summarized in Table 1. The median age in the population was 67 years old (63–69), 50% of patients had an ECOG (Eastern Cooperative Oncology Group) performance status (PS) >1 and 32% had brain metastases at the initiation of nivolumab. The most frequent histological tumor type was adenocarcinoma (71%). Half of the patients initially received at least two lines of treatment before nivolumab initiation, including tyrosine kinase inhibitor (14%) or bevacizumab (39%). Lymphopenia before nivolumab starts was observed in eight patients (29%). Three patients (11%) definitively discontinued nivolumab therapy due to severe toxicities: grade 4 diarrhea (*n* = 1); grade 3 dyspnea and hypercalcemia (*n* = 1) and grade 3 dyspnea with pulmonary hypertension (*n* = 1). At data cut-off, 6 patients (21%) were still treated with nivolumab.

### 2.2. Basal Kinomic Activity Profiles in NSCLC Patients

**Figure 1:**
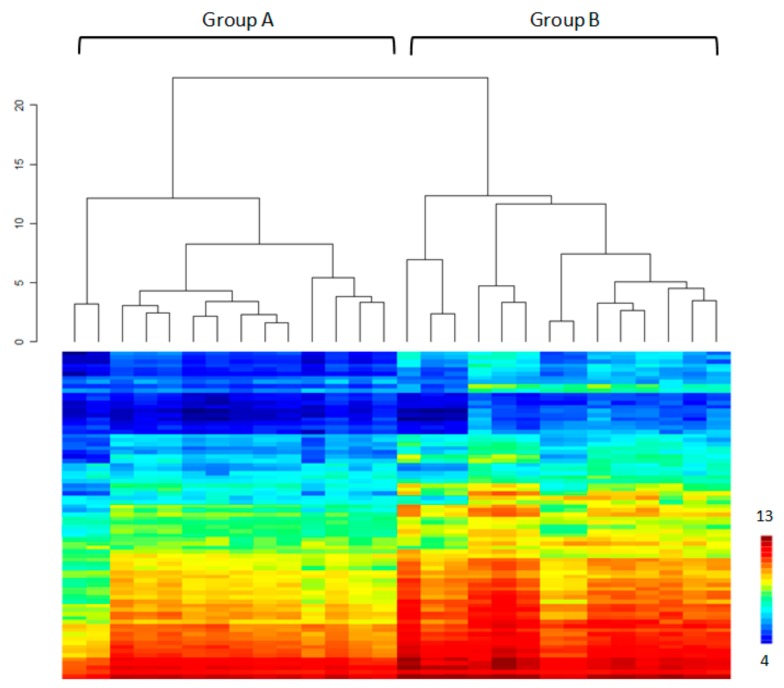
Unsupervised hierarchical clustering of basal kinase activity profiles among non-small-cell lung cancer patients peripheral blood mononuclear cells (PBMC). The heatmap represents phosphorylation levels (Log2Signal) for 80 peptides (Y-axis) sorted by hierarchical clustering. Two distinct clusters can be observed among the 28 patients’ PBMC.

After applying the nominal coefficient variation (CV) threshold, there were only 80 STK substrates left: “QC list” (see Supplementary Table S1). Basal STK kinomic profiles in PBMC from NSCLC patients, obtained using microarrays before nivolumab starts (D0), could be divided into two major clusters (clusters A and B) after applying a hierarchical clustering (Figure 1, Supplementary Table S2). Kinomic profiles of patients from clusters A and B were compared using a Student’s *t*-test. Almost all peptides (79 with *p* < 0.05) showed significantly lower phosphorylation levels in cluster A. Amongst the putative upstream STK analysis we found less activity in ZC1/2/3/4, NEK4, CDK, DAPK kinases in PBMC from patients of cluster A. In order to explain the difference in kinomic profiles between clusters A and B, different baseline parameters (demographical, clinical and biological) were compared (Table 2). Interestingly, the PD-L1 tumor cell (TC) score (corresponding to expression of PD-L1 evaluated by immunohistochemistry on tumor cells from pretreatment tumor biopsy) (*p* = 0.045), the count of CD8+ cells (*p* = 0.023) and the total body weight (*p* = 0.038) were statistically different between the two clusters.

### 2.3. Comparison between Baseline Kinomic Profiles from Healthy Volunteers and NSCLC Patients

Basal STK activity in PBMC from 12 healthy volunteers were also assessed using kinomic approach. Median age was 50 (48–57) years in healthy volunteers, and half of them were male.

**Figure 2:**
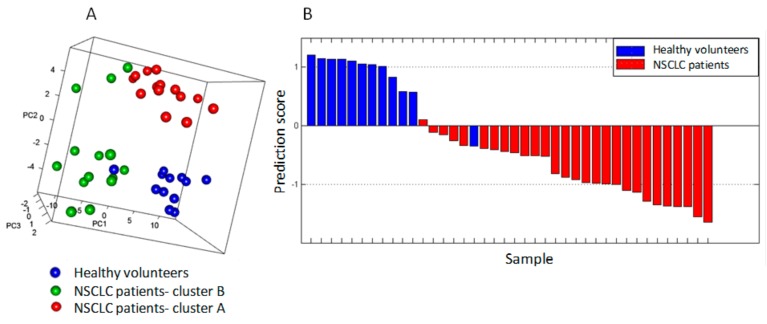
Prediction of PBMC source based on basal serine/threonine kinase (STK) activity profiles. (**A**) The 3D plot, obtained after applying unsupervised principal component analysis (PCA), separates samples in three groups. Each label represents a PBMC sample from 12 healthy volunteers (blue) or non-small-cell lung cancer (NSCLC) patients: 14 from cluster A (red) and 14 from cluster B (green). All volunteers, except one, remain in the same group. (**B**) Partial least squares discriminant analysis classifies healthy volunteers (blue) and patients (red) PBMC. Samples prediction scores were obtained based on a leave-one-out cross-validation. Samples with a positive score correspond to predicted healthy volunteers whereas samples with a negative score correspond to predicted NSCLC patients.

Unsupervised principal component analysis (PCA) showed good separation between healthy volunteers and NSCLC patients (see Supplementary Figure S1). In contrast with healthy volunteers, a large interindividual variability in kinomic activity profiles was observed in NSCLC patients (Figure 2A). Additionally, NSCLC patients in cluster A presented basal STK activity profiles close to those of healthy volunteers. The supervised analysis using partial least squares-discriminant analysis (PLS-DA), evaluated by 20-fold cross-validation, classified healthy volunteers and NSCLC patients based on the basal STK kinomic profiles. Samples from NSCLC patients and healthy volunteers were correctly classified in 96% and 92% of cases, respectively (Figure 2B).

Furthermore, a Student’s *t*-test was applied to compare healthy volunteers’ STK kinomic profiles to those of patients. Majority of peptides were significantly different between these two groups (67 peptides with *p* < 0.05; 62 with *p* < 0.01), with a significantly lower phosphorylation level for 75% of peptides in healthy volunteers. Kinexus Kinase Predictor was used to determine putative upstream STK. Among the top 15 STK which were different between the two groups, many of them belong to the CAMK family and AGC family. The top three kinases most altered were: VACAMKL, DCAMKL, CaMK2 and Akt (1/2/3), PKCε, PKACγ, for CAMK and AGC family, respectively.

### 2.4. Inhibition Profiles in NSCLC Patients at Day 14

Differences in peptides phosphorylation before and after nivolumab therapy obtained after a Student paired *t*-test are summarized in Supplementary Table S3.

**Figure 3:**
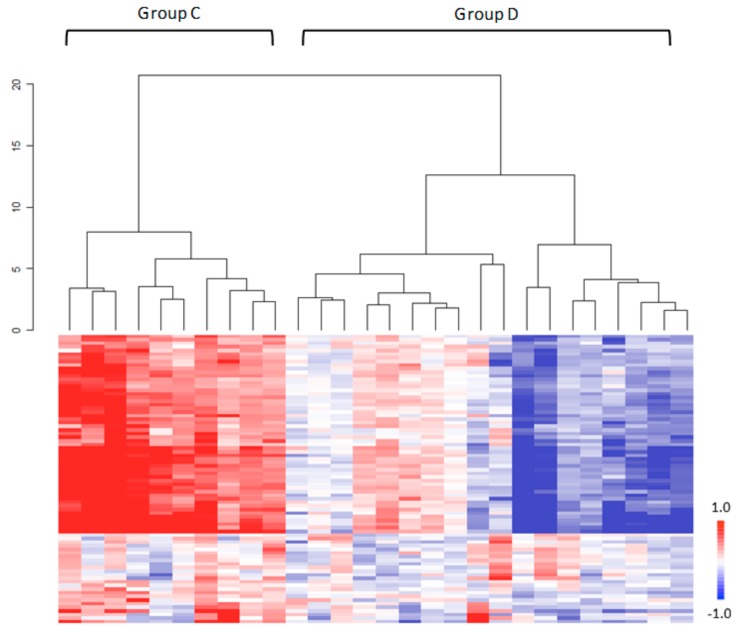
Inhibition profiles in PBMC from non-small-cell lung cancer patients under nivolumab therapy. Unsupervised hierarchical clustering, using Log fold change (LFC) values (Log_2_Signal (D14)−Log_2_Signal (D0)) of 80 STK substrates (*Y*-axis), separates NSCLC samples (*n* = 28) in two major clusters. Red color represents a low inhibition level, whereas blue corresponds to a strong inhibition.

To evaluate the pharmacological effect of nivolumab on PBMC, the ratios of peptides phosphorylation at day 14 (D14) of the first nivolumab administration and D0 (Log_2_ fold change, LFC = Log_2_Signal at D14−Log_2_Signal at D0) were investigated in the 28 NSCLC patients just prior to the second infusion of nivolumab. A large interindividual variability in kinomic profiles was observed (Figure 3). Unsupervised hierarchical clustering identified two major clusters (cluster C and cluster D), with two subsets within the cluster D (Figure 3, Supplementary Table S2). Cluster C presents activation profiles whereas a part of cluster D is composed of strong inhibition profiles. The comparison of clusters C and D showed a statistical difference for 66 peptides (*p* < 0.05). Akt, CAMK, NEK, AurA/C, PLK, PKC kinases were found to be involved in the difference observed between cluster C and cluster D profiles.

The proportion of patients from cluster A were equally distributed in both clusters C and D. No significant variation in biochemical and hematological parameters was observed over time. Interestingly, 56% of patients in cluster D were previously treated with bevacizumab, and none in cluster C (*p* = 0.011). Additionally, patients in cluster D had significantly more (2.5-fold) CD8+ T lymphocytes (339 vs. 140 cells/mm^3^, respectively; *p* = 0.05) (Table 3).

### 2.5. Relationship between Efficacy and Kinomic Profile

**Figure 4:**
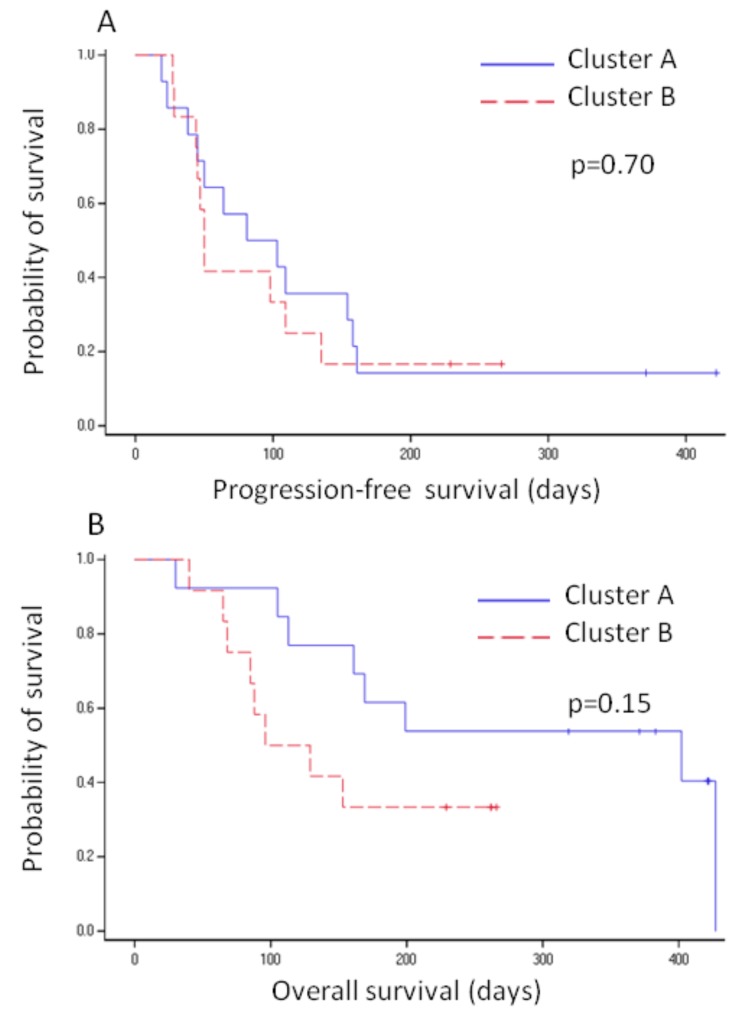
Kaplan-Meier curves of the (**A**) progression-free survival and (**B**) overall survival proportion according to basal kinomic profile. Blue and red lines indicate patients in clusters A and B, respectively. Small crosses represent censored patients. Median progression free survival (PFS) was 92 (38–158) and 50 (28–135) days in clusters A and B, respectively (log-rank test *p*-value; *p* = 0.70). Median OS was 402 (113–427) and 112.5 (65-not determined) days in clusters A and B, respectively (log-rank test *p*-value; *p* = 0.15).

Two and three patients lost to follow-up were excluded from the analysis of the relationship between kinomic profiles and progression free survival (PFS) or overall survival (OS) relationship, respectively. The median PFS (*n* = 26) and OS (*n* = 25) in the whole cohort were 72.5 (confidence interval 95%, (95% CI) 45–109) and 169 (95% CI, 105–427) days, respectively. Regarding the basal kinomic profiles, PFS was similar in clusters A and B (median: 92 (38–158) vs. 50 (28–135) days, respectively; *p* = 0.70) (Figure 4A). Interestingly, patients in cluster A tended to have a longer median OS than those in cluster B (402 (113–427) vs. 112.5 (65-not determined) days, respectively; *p* = 0.15) (Figure 4B). The OS rate at 1 year was 62% (95% CI, 35–88) in cluster A vs. 33% (95% CI, 7–60) in cluster B (*p* = 0.12). Regarding the kinomic profile on D14, no significant difference was observed between clusters C and D for PFS (103 (19–161) vs. 50 (38–135) days, respectively; *p* = 0.87) and OS (165 (30–427) vs. 199 (65-not determined) days, respectively; *p* = 0.92).

## 3. Discussion

This study presents the different kinomic profiles among in PBMC from healthy volunteers and NSCLC patients, prior to nivolumab initiation and after the first injection. We identified two clusters of PBMC kinomic profile at D0 among NSCLC patients, which differed from healthy volunteers’ profiles, and demonstrated that nivolumab therapy induces substantial changes in STK activities, particularly in those involved in phosphoinositide 3-kinase (PI3K/Akt) pathway. To the best of our knowledge, this work is the first to assay the kinomic profile in PBMC as a pharmacodynamic biomarker in NSCLC patients treated with nivolumab and to characterize the pharmacological effect of nivolumab on STK kinomic profile. Baseline cluster A kinomic profile seems to correlate with overall survival, although this result did not reach statistical significance in this limited population. Our results confirm the necessity to identify a specific prognostic biomarker since nivolumab is not a treatment that fits for all patients, especially for those from the real-life population. The present study describes a less favorable outcome in terms of survival than those reported in two phase III trials [5,6]. The patients included in our study were more fragile than those included in phase III trials since they exhibit poorer ECOG PS (50% of ECOG PS >1) and more frequently brain metastases (32%) at the initiation of nivolumab therapy. The assessment of PD-L1 expression in the stromal compartment as a biomarker for the clinical use of nivolumab in NSCLC patients currently remains controversial [20]. Recently, an immunoscore including the expression of PD-L1+ immune cells in the stromal compartment and PD-L1+ intraepithelial tumor-infiltrating lymphocytes was proposed as independent positive prognostic factors for NSCLC patients [21]. However, these two approaches are based on pre-treatment tumor biopsy, which may fail to reflect current tumor dynamics and variation of drug sensitivity over the treatment course as recently suggested [22]. Nivolumab mainly targets T cells; however, isolation of T cells requires an additional step (immunomagnetic isolation method or a flow cytometric immunophenotyping) which could alter kinase activity. Furthermore, this additional isolation step would be time-consuming. In this context, PBMC could be a perfect surrogate tissue for the identification and assessment of pharmacodynamic biomarkers, as this tissue is readily accessible for repeated sampling throughout therapy.

We identified in baseline two clusters A and B, with a 4-fold longer median OS in the cluster A. However, the difference in OS between the two clusters did not reach the statistical significance (*p* = 0.15), probably because of the limited size of our cohort. Interestingly, the kinomic profile in cluster A was closed to that of healthy volunteers. Additionally, the baseline number of CD8+ T cells in cluster A was within the normal range (150 to 1000 cells/mm^3^) in contrast with cluster B, in which it was approximately three-fold lower. This difference could contribute to an enhanced infiltration of these cytotoxic cells in Cluster A tumor microenvironment during nivolumab therapy and therefore favor better outcomes. Interestingly, a higher PD-L1 expression on tumor cells in cluster B was associated with a stronger STK activity in PBMC from NSCLC patients (cluster B vs. A, *p* = 0.045). Chevolet et al. demonstrated that PD-L1 was equally expressed in leukocyte subsets in blood and tumor bed from melanoma patients [23]. Moreover, they demonstrated that increased PD-L1 expression in cytotoxic T cells was associated with worse OS. Jacquelot et al. recently observed that elevated PD-L1 expression on peripheral T cells correlated with ex vivo resistance to CTLA4 blockade in advanced melanoma [24]. Different studies showed that some peripheral blood parameters were associated with clinical outcomes in lung cancer patients treated with nivolumab. Thus, increased baseline neutrophils count, decreased baseline lymphocytes count, increased neutrophil-to-lymphocyte ratio and increased LDH are associated with poorer survival [25,26]. In fact, baseline LDH level and NLR are prognostic factors for lung cancer patients regardless of the anticancer agent [27]. In our study, there was no difference for these parameters either between clusters A and B or between clusters C and D. This observation raises the hypothesis that difference between clusters A and B could be rather associated with different cell signaling pathways activation than a different prognosis. Overall, further investigations with larger cohorts are warranted to investigate the benefit of basal kinomic profile and PD-L1 expression in PBMC to predict therapeutic response to nivolumab.

On D14, two distinct clusters (C and D) were identified. The activity of most STK was higher in cluster C, especially those of the (PI3K)/Akt signaling pathway. The plasma trough concentration of nivolumab on D14 was similar in the two clusters, suggesting that plasma drug exposure could not explain the interindividual variability in kinomic profiles. In the same way, basal kinomic profile of NSCLC patients cannot contribute to the observed variability in the pharmacological effect of nivolumab since the proportion of patients from cluster A were equally distributed in both clusters C and D. Interestingly, 56% of patients in cluster D were previously treated with bevacizumab, and none in cluster C (*p* = 0.011). Bevacizumab is a monoclonal antibody which specifically binds to VEGF-A in the systemic circulation. Different studies showed that VEGF-A enhances the expression level of PD-1 on lymphocytes [28,29]. This suggests higher PD-1 expression in cluster C than in cluster D. Jacquelot et al. have recently reported an association between a greater baseline level of PD-1 (>20%) on circulating CD4+ T cells from melanoma patients and the likelihood to respond to an ex vivo metastatic lymph nodes assay using anti-PD1 monoclonal antibody [24]. Besides, engaged PD-1 (i.e., interaction between PD-1 and its ligand PD-L1) inhibits PI3K/Akt signaling pathway [30,31]. In this context, an increased expression of PD-1 on PBMC from patients of cluster C could result in enhanced pharmacological effect of nivolumab, expressed through an increase in PD-1/PD-L1 interaction blockage by nivolumab, and thereby restore a greater activity of kinases involved in the PI3K/Akt signaling pathway in patients’ PBMC. However, the difference in pharmacodynamic profiles of clusters C and D did not enable to discriminate responders from nonresponders. Voron et al. showed that blocking VEGF-A/VEGFR axis decreases PD-1 expression on intratumoral CD8+ T cells and restores IFN-γ production in intratumoral CD8+ T cells [28] which is known to promote tumor growth [32]. Given that 56% of patients in cluster D were previously treated with bevacizumab and none in cluster C (*p* = 0.011), this heterogeneity in the two clusters could provide a bias in the statistical analysis, since the number of peripheral CD8+ lymphocytes was statistically higher in cluster D. Thus, further investigations to assess the benefit of kinomic profiling on D14 in a homogeneous population regarding pre-treatment with bevacizumab should be considered.

We observed different basal kinomic profiles of PBMC from NSCLC patients compared to healthy volunteers. Additionally, we could classify samples from healthy volunteers and NSCLC on the basis of their kinomic profile in 92% and 96% of cases, respectively. The activity of most STK was higher in NSCLC patients, especially those of the PI3K/Akt signaling pathway. Most of NSCLC patients previously received chemotherapy agents such as platinum compounds, etoposide or paclitaxel. These chemotherapy agents are known to induce PI3K/Akt pathway in tumor [33]. Additionally, Yan et al. recently showed that exposure of T-leukemia/lymphoma cells or Jurkat T cells to cisplatin upregulated miR181a expression and resulted in Akt activation [34]. In this context, one hypothesis could be that the previous chemotherapy treatment would contribute to the increased Akt activation in NSCLC patients compared to healthy volunteers. Interestingly, Li et al. showed that persistent antigen (Ag) exposure results in the activation of the Akt/mTORC1 pathway in Ag-specific CD8+ T cells [35]. In this context, one cannot exclude that continuous exposure to tumor neoantigens could also contribute to this sustained activation of PI3K/Akt signaling pathway in NSCLC patients.

These results present two limits. First, the groups of healthy volunteers and NSCLC patients were not matched according to sex and age because including healthy elderly volunteers without comorbidities and not under medications remains a challenge. Second, our kinomic results were not confirmed using western blot. This confirmation step should be encouraged for the next studies including kinomic approach. In the future, it would be interesting to analyze the kinomic profile in PBMC from naïve-treatment NSCLC patients in order to know whether the kinomic approach could discriminate NSCLC patients from healthy volunteers.

## 4. Methods

### 4.1. Population and Study Design

From August 2015 to May 2016, 12 healthy volunteers and 28 consecutive outpatients treated with single-agent nivolumab for a metastatic NSCLC in the oncology department of Cochin Hospital in Paris, France, were prospectively analyzed. Patients were >18 years-old, previously treated with chemotherapy and/or targeted therapy (tyrosine kinase inhibitors or bevacizumab) and with ECOG PS ≤2. Patients with autoimmune disease were excluded. Blood samples for every patient were collected before treatment initiation (D0) and just before the second nivolumab infusion (D14). Biochemical parameters (albumin, C-reactive protein (CRP), lactate dehydrogenase) and hematological parameters including subset analyses of peripheral lymphocytes were determined on D0 and D14. Additionally, the concentration of nivolumab in plasma was assayed on D14. Patients were clinically evaluated every 2 weeks. Tumor PD-L1 protein expression (PD-L1 Tumor Cell score) was evaluated retrospectively in pretreatment (archival or recent) tumor-biopsy or surgical resection specimens with the use of an automated immunohistochemistry assay, that used rabbit monoclonal anti-human PD-L1 antibody (E1L3N Cell Signaling Technology—automat Leica, Nanterre, France).

### 4.2. Ethics Statement

This prospective study was approved by the local medical ethical board: CLEC (local ethics committee in oncology) (N°286615) and all subjects provided written informed consent and agreed to the sampling and kinomic analysis in accordance with the Declaration of Helsinki.

### 4.3. Treatment Schedule

All NSCLC patients were treated with the recommended dose of nivolumab (3 mg/kg every two weeks). Nivolumab-related adverse events were graded according to the National Cancer Institute Common Terminology Criteria for Adverse Events (NCI-CTCAE) version 4.0. Nivolumab administration was continued until disease progression, intolerable adverse events or patient refusal. In the case of grade ≥2 toxicity, the treatment was discontinued until adverse events returned to baseline if needed corticosteroids could be used to help resolution. In the case of grade 4 toxicity (or grade 3 pneumonitis, colitis and adrenal insufficiency), nivolumab was permanently discontinued.

### 4.4. Efficacy Assessment

Tumor response was assessed with the use of imaging after 2 months of treatment, and then every 2 months. PFS and OS were defined as the time from the first infusion of nivolumab to the date of disease progression or any cause of death for the former, and the date of death for the latter. In case of radiological progression (according to RECIST 1.1) without clinical worsening at two months, second imaging after 4 weeks was performed to confirm the first one.

### 4.5. Nivolumab Concentration Assay in Plasma

Trough plasma concentration of nivolumab was determined on D14 (just prior to the second infusion) using an ELISA method, as previously described by our group [36]. The intra- and inter-assay coefficients of analytical variability were less than 5% and 12%, respectively. The lower limit of quantification was 3 µg/mL.

### 4.6. Sample Collection and Processing for Kinomic Profiling

Whole blood samples (10 mL) were collected in healthy volunteers (*n* = 12) with no chronic disease or concomitant treatment, and in patients (at D0 and D14). Then, PBMC were isolated and stored at −80 °C until kinomic analysis, as previously described [13].

Protein quantification was performed using standard Bradford protein assay (Thermo Scientific). For each experiment 1 µg of protein lysate was added to the assay mixture, containing 400 µM ATP and primary STK antibody solution (PamGene International B.V.’s, Hertogenbosch, The Netherlands). The mixture was loaded onto STK PamChip^®^. The latter contains four identical arrays where 144 synthesized phosphorylatable peptides (13 amino acids long), substrates of STK, are immobilized on a porous membrane. The mixture was pumped through the arrays to enhance interactions between peptides and lysates’ STK. After a washing step, a mixture of secondary antibody FITC-labeled (fluorescein isothiocyanate) (PamGene) was used for visualization.

STK activity profiling was performed using a PamStation^®^12 (PamGene). Peptide phosphorylation was recorded after adding the secondary antibody by assessing fluorescence with a CCD camera in combination with Evolve software v. 1.2 (PamGene) at different exposure times (20, 50, 100 and 200 ms). 

### 4.7. Statistical Analysis

#### 4.7.1. Kinomic Approach

Image analysis and signal quantification were performed using the BioNavigator^®^ software v. 6.1 (PamGene) interfaced with R (Bioconductor, Hertogenbosch, The Netherlands). All peptides with low signal were removed using the variation of biological replicates. Peptides presenting nominal CV lower than 50% were preselected (“QC list”) and Log_2_-transformed (“Log_2_Signal”). Log fold change (LFC) was obtained by making the difference between D14 Log_2_Signal and D0 Log_2_Signal. Unsupervised hierarchical analysis, using Euclidean distance metrics and complete linkage, of peptide signals (Log_2_Signal or LFC) was performed and displayed as heatmaps. Kinexus Kinase Predictor was used to determine putative upstream STK from the peptides lists within the clusters. Student’s *t*-tests were used to compare healthy and patients profiles. Unsupervised multivariate clustering of samples was evaluated with principal component analysis (PCA). Supervised multivariate analysis of conditions was conducted by applying partial least squares discriminant analysis (PLS-DA) in Matlab Statistics Toolbox (MathWorks, Naticke, MA, USA) implemented in the BioNavigator^®^. Prediction performance was evaluated by 20-fold cross-validation to ensure that the model was optimized completely independent of the test sample.

#### 4.7.2. Survival Endpoint

Descriptive statistics used numbers (percentages) for qualitative variables and median (inter-quartile intervals) for quantitative ones. Evolutions of biological parameters were analyzed using repeated measure ANOVA, followed by Tukey’s tests for pairwise comparisons when the global test was significant. Comparisons between two clusters were tested using Fisher’s exact tests for qualitative variables and Mann-Whitney-Wilcoxon tests for quantitative ones. PFS and OS were analyzed in the framework of survival analysis. Thus, the PFS and OS functions were estimated by the Kaplan-Meïer method. All computations were performed using the SAS v 9.4 statistical package (SAS Institute Inc., Cary, NC, USA).

## 5. Conclusions

In conclusion, this work identifies two different basal STK kinomic profiles of PBMC in NSCLC patients before the initiation of nivolumab therapy. Although not statistically significant, one cluster exhibited a 4-fold longer median OS. This finding paves the way to further investigate a larger cohort of NSCLC patients for the benefit of the basal STK kinomic profile in PBMC to identify responders to nivolumab therapy.

## Figures and Tables

**Table 1:** Demographic and baseline characteristics of patients.

Characteristics	*n* = 28
Demographic data	
Sex	
Male	17 (61%)
Female	11 (39%)
Age (years) Age > 70 years-old	67 (63–69) 6 (21%)
BMI (kg/m^2^) Lean Body mass (kg)	22.9 (19.9–24.1) 49.4 (42.2–56.4)
Smoking status	
Current smoker	6 (21%)
Former smoker	17 (61%)
Never smoker	3 (11%)
Not available	2 (7%)
ECOG performance status	
0–1	14 (50%)
2	14 (50%)
**Non-small-cell lung cancer characteristics**	
Histological tumor type	
Squamous cell carcinoma	8 (29%)
Adenocarcinoma	20 (71%)
Metastasis Synchronous Metachronous Initial surgery Yes No Number of previous treatment lines 1 2 >2	19 (68%) 9 (32%) 10 (36%) 18 (64%) 14 (50%) 8 (29%) 6 (21%)
Previous Targeted Therapy	
Tyrosine Kinase Inhibitor ^a^	4 (14%)
Monoclonal antibody (bevacizumab)	11 (39%)
None	14 (50%)
Number of metastatic sites	
1	4 (14%)
2	15 (54%)
≥3	9 (32%)
Cerebral metastasis	
Yes	9 (32%)
No	19 (68%)
**Baseline Biological Data**	
Haemoglobin (g/dL)	12.9 (11.6–14.3)
Platelets (×10^9^/L)	231 (204–298)
Lymphocytes (×10^9^/L)	1.26 (0.98–1.65)
Neutrophils (×10^9^/L)	5.38 (4.28–6.32)
Lymphopenia (<1 × 10^9^/L) before Nivolumab, *n* (%) LDH Increased above ULN Normal Non available	8 (29%) 12 (43%) 10 (36%) 6 (21%)

BMI, body mass index; ECOG, Eastern Cooperative Oncology Group; LDH, lactate dehydrogenase; ULN upper limit of normal. ^a^ erlotinib (*n* = 3), crizotinib (*n* = 1). Results are expressed as median (interquartile range) or number of patients (percent %).

**Table 2:** Characteristics of the two clusters A and B of non-small-cell lung cancer patients identified before treatment start (baseline).

Characteristics	Cluster A (*n* = 14)	Cluster B (*n* = 14)	*p* Value
Sex (female) (%)	50.0	71.4	0.44
Age (years old)	66 (63–74)	68 (62–69)	1.0
Total body weight (kg)	65 (53–68)	71 (62–74)	**0.038**
ECOG performance status			0.26
0–1	9 (64%)	5 (36%)	
>1	5 (36%)	9 (64%)	
Current/former Smoker (%)	77.0	100.0	0.22
Histological tumor type			1.0
Adenocarcinoma	10 (71.4%)	10 (71.4%)	
Squamous cell carcinoma	4 (28.6%)	4 (28.6%)	
PD-L1 TC score	0.5 (0–5)	10 (1.5–75)	**0.045**
Presence of molecular alteration * (%)	36.4	27.3	1.0
Time to metastasis (days)	0 (0–243)	0 (0–273)	0.74
Number of metastatic sites			1.0
1–2	10 (71.4%)	9 (64.3%)	
>2	4 (28.6%)	5 (35.7%)	
Number of previous treatment line			0.71
1–2	6 (42.9%)	8 (57.1%)	
>2	8 (57.1%)	6 (42.9%)	
Previous targeted therapy			
Tyrosine kinase inhibitor (%)	21.4	7.4	0.59
Bevacizumab (%)	36.0	43.0	1.0
Corticosteroids treatment (%)	14.3	21.4	1.0
**Baseline biological characteristics**			
C reactive protein (mg/L)	10.4 (1.3–15.4)	5.4 (3.2–31.9)	0.73
LDH (UI)	218 (193–267)	210 (197–303)	0.84
Albumin (g/L)	42 (40–45)	38 (36–42)	0.07
Neutrophils count (cells/mm^3^)	5180 (3820–6310)	5850 (5160–6350)	0.57
Lymphocytes count (cells/mm^3^)	1510 (1050–2020)	1140 (610–1460)	0.09
Neutrophils-lymphocytes ratio (NLR)	4.1 (2.3–5.4)	5.1 (3.6–7.0)	0.23
CD3+ cells count (cells/mm^3^)	1069 (750–1402)	822 (447–959)	0.14
CD4+ cells count (cells/mm^3^)	701 (301–914)	460 (235–640)	0.17
CD8+ cells count (cells/mm^3^)	365 (218–439)	137 (95–327)	**0.023**
B cells count (cells/mm^3^)	114 (88–204)	92 (34–170)	0.13
NK count (cells/mm^3^)	129 (82–145)	101 (64–153)	0.47
Proportion of CD3+ cells (%)	77.9 (70.9–85.6)	84.2 (76.7–90.8)	0.22
Proportion of CD4+ cells (%)	43.5 (36.6–54.7)	61.9 (35.2–69.2)	0.21
Proportion of CD8+ cells (%)	27.8 (17.9–35.5)	18.0 (10.4–36.7)	0.18
Proportion of NK cells (%)	9.2 (5.0–14.0)	9.6 (6.1–15.7)	0.66
Proportion of B cells (%)	11.2 (6.6–21.9)	10.6 (7.7–13.0)	0.56

ECOG, Eastern Cooperative Oncology Group; LDH, lactate dehydrogenase; NK, natural killer; PD-L1 TC, programmed death ligand 1 tumor cell. * molecular alteration of ALK, EGFR, KRAS or HER2. Results are expressed as median (interquartile range) or number of patient (percent%). In bold, *p* value less than 0.05. Proportion of immune cells were determined by immunophenotyping, using a flow cytometry protocol.

**Table 3:** Characteristics of the two clusters C and D of non-small-cell lung cancer patients identified after a single administration of nivolumab (14 days after treatment start).

Characteristics	Cluster C (*n* = 10)	Cluster D (*n* = 18)	*p* Value
Sex (female) (%)	70.0	55.5	0.69
Age (years old)	68 (64–70)	65 (62–69)	0.43
Total body weight (kg)	68 (61–71)	68 (54–73)	0.90
ECOG performance status			0.24
0–1	3 (30%)	11 (61.1%)	
>1	7 (70%)	7 (38.9%)	
Current/former Smoker (%)	88.9	88.2	1.0
Histological tumor type			0.40
Adenocarcinoma	6 (60.0%)	14 (77.8%)	
Squamous cell carcinoma	4 (40.0%)	4 (22.2%)	
Presence of molecular alteration * (%)	33.3	31.3	1.0
Time to metastasis (days)	0 (0–92)	0 (0–273)	0.82
Number of metastatic sites,			0.42
1–2	8 (80%)	11 (61.1%)	
>2	2 (20%)	7 (38.9%)	
Number of previous treatment line,			1.0
1–2	5 (50%)	9 (50%)	
>2	5 (50%)	9 (50%)	
Previous targeted therapy			
Tyrosine kinase inhibitors (%)	20.0	11.1	0.60
Bevacizumab (%)	0	55.6	**0.011**
Corticosteroids treatment (%)	0	27.8	0.13
**Biological characteristics on day 14**			
Plasma concentration of nivolumab (mg/L) on day 14	15.8 (13.0–22.9)	15.9 (12.4–18.8)	0.58
C reactive protein (mg/L)	6.5 (1.6–19.9)	11.9 (5.3–28.3)	0.40
LDH (UI)	265 (198–305)	198 (168–244)	0.28
Albumin (g/L)	39 (36–41)	36 (34–40)	0.25
Neutrophils count (cells/mm^3^)	5160 (4580–5870)	4960 (4560–5980)	0.92
Lymphocytes count (cells/mm^3^)	990 (860–1230)	1330 (790–1650)	0.41
Neutrophils-lymphocytes ratio (NLR)	4.25 (3.60–7.80)	4.20 (3.20–6.20)	0.71
CD3+ cells count (cells/mm^3^)	702 (637–846)	894 (503–1222)	0.29
CD4+ cells count (cells/mm^3^)	541 (394–618)	609 (207–806)	0.76
CD8+ cells count (cells/mm^3^)	140 (102–288)	339 (195–453)	**0.05**
B cells count (cells/mm^3^)	92 (59–122)	90 (59–184)	1.0
NK count (cells/mm^3^)	113 (100–160)	106 (68–177)	0.66
Proportion of CD3+ cells (%)	81.3 (75.2–94.4)	80.1 (60.6–88.7)	0.76
Proportion of CD4+ cells (%)	52.8 (43.4–59.4)	43.0 (31.9–56.4)	0.18
Proportion of CD8+ cells (%)	17.0 (14.6–27.0)	29.4 (17.9–42.1)	0.11
Proportion of NK cells (%)	11.1 (8.5–15.5)	9.8 (5.5–16.2)	0.50
Proportion of B cells (%)	10.2 (5.9–11.7)	8.3 (5.9–14.6)	0.54

ECOG, Eastern Cooperative Oncology Group; LDH, lactate dehydrogenase; NK, natural killer. *molecular alteration of ALK, EGFR, KRAS or HER2 kinases. Results are expressed as median (interquartile range) or number of patients (percent%). In bold, *p* value less than 0.05. Proportion of immune cells were determined by immunophenotyping, using a flow cytometry protocol.

## Supplementary Materials

The following are available online at https://www.mdpi.com/2072-6694/11/6/762/s1. Figure S1: Unsupervised hierarchical clustering of basal kinase activity profiles among non-small-cell lung cancer patients PBMC (*n* = 28) and healthy volunteers (*n* = 12), Table S1: QC list of the 80 peptides substrates of serine/threonine kinases presenting nominal CV lower than 50%, Table S2: QC list peptides (*n* = 80) ranking within the different clusters obtained after unsupervised hierarchical clustering of the kinase activity profiles. Peptides ordering is related to Figure 1 and Figure 3, Table S3: Differences in peptides phosphorylation (*n* = 80) before and after nivolumab treatment, determined by a Student’s paired *t*-test.
