# Supplementary material for: Differential Kinase Activation in Peripheral Blood Mononuclear Cells from Non-Small-Cell Lung Cancer Patients Treated with Nivolumab
Source: Cancers (Basel). 2019 May 31;11(6):762. doi: 10.3390/cancers11060762 (PMC6628172; doi:10.3390/cancers11060762)
Supplement: Supplementary file 1 [file cancers-11-00762-s001.pdf]

# Supplementary Materials: Differential Kinase Activation in Peripheral Blood Mononuclear Cells from Non-Small Lung Cancer Patients Treated with Nivolumab

**Gaëlle Noé, Audrey Bellesoeur, Lisa Golmard, Audrey Thomas-Schoemann, Pascaline Boudou-Rouquette, Manuela Tiako Meyo, Alicja Puszkiel, Jennifer Arrondeau, Jérome Alexandre, François Goldwasser, Benoit Blanchet and Michel Vidal**

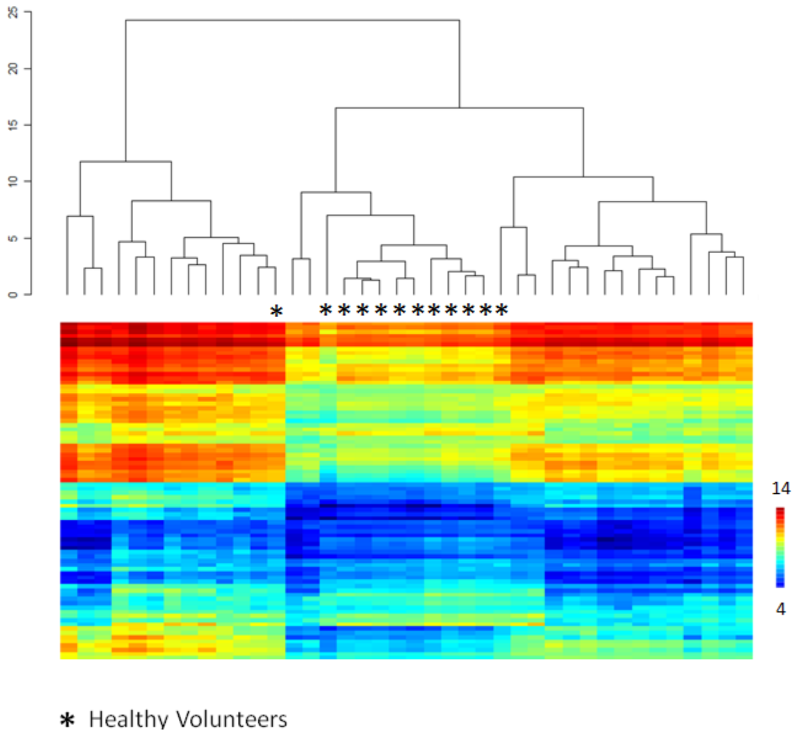


**Figure S1.** Unsupervised hierarchical clustering of basal kinase activity profiles among non-small-cell lung cancer patients PBMC (*n* = 28) and healthy volunteers (*n* = 12). The heatmap represents phosphorylation levels ($Log_2$Signal) for 80 peptides of the "QC List" (*Y*-axis) sorted by hierarchical clustering. Patients are well separated from healthy volunteers; patients are clearly classified in two distinct clusters.

**Table S1.** QC list of the 80 peptides substrates of serine/threonine kinases presenting nominal CV lower than 50%.

| Rank | QC List (in Alphabetical Order) | Rank | QC List (in Alphabetical Order) |
|---|---|---|---|
| **1** | ACM1_421_433 | **41** | KCNA1_438_450 |
| **2** | ACM4_456_468 | **42** | KCNA2_442_454 |
| **3** | ACM5_494_506 | **43** | KCNA3_461_473 |
| **4** | ACM5_498_510 | **44** | KCNA6_504_516 |
| **5** | ADDB_706_718 | **45** | KIF2C_105_118_S106G |
| **6** | ADRB2_338_350 | **46** | KPB1_1011_1023 |
| **7** | ANDR_785_797 | **47** | KPCB_19_31_A25S |
| **8** | ANXA1_209_221 | **48** | KS6A1_374_386 |
| **9** | ART_025_CXGLRRWSLGGLRRWSL | **49** | LIPS_944_956 |
| **10** | BAD_112_124 | **50** | MARCS_160_172 |
| **11** | BAD_69_81 | **51** | MBP_222_234 |
| **12** | BAD_93_105 | **52** | MP2K1_287_299 |
| **13** | CA2D1_494_506 | **53** | MPIP1_172_184 |
| **14** | CAC1C_1974_1986 | **54** | MYPC3_268_280 |
| **15** | CDN1A_139_151 | **55** | NCF1_296_308 |
| **16** | CENPA_1_14 | **56** | NCF1_321_333 |
| **17** | CFTR_730_742 | **57** | NFKB1_330_342 |
| **18** | CFTR_761_773 | **58** | NMDZ1_890_902 |
| **19** | CGHB_109_121 | **59** | NOS3_1171_1183 |
| **20** | CREB1_126_138 | **60** | NR4A1_344_356 |
| **21** | CSF1R_701_713 | **61** | PLEK_106_118 |
| **22** | DESP_2842_2854 | **62** | PLM_76_88 |
| **23** | E1A_ADE05_212_224 | **63** | PPR1A_28_40 |
| **24** | EPB42_241_253 | **64** | PTK6_436_448 |
| **25** | ERBB2_679_691 | **65** | PTN12_32_44 |
| **26** | ESR1_160_172 | **66** | RAP1B_172_184 |
| **27** | F263_454_466 | **67** | RBL2_655_667 |
| **28** | FOXO3_25_37 | **68** | RB_242_254 |
| **29** | FRAP_2443_2455 | **69** | RB_803_815 |
| **30** | GBRB2_427_439 | **70** | REL_260_272 |
| **31** | GPR6_349_361 | **71** | RS6_228_240 |
| **32** | GPSM2_394_406 | **72** | RYR1_4317_4329 |
| **33** | GRIK2_708_720 | **73** | SCN7A_898_910 |
| **34** | GYS2_1_13 | **74** | STK6_283_295 |
| **35** | H2B1B_ 27_40 | **75** | STMN2_90_102 |
| **36** | H32_3_18 | **76** | TOP2A_1463_1475 |
| **37** | K6PL_766_778 | **77** | TY3H_65_77 |
| **38** | KAP2_92_104 | **78** | VASP_150_162 |
| **39** | KAP3_107_119 | **79** | VASP_271_283 |
| **40** | KAPCG_192_206 | **80** | VTNC_390_402 |

**Table S2.** QC list peptides (*n* = 80) ranking within the different clusters obtained after unsupervised hierarchical clustering of the kinase activity profiles. Peptides ordering is related to Figures 1 and 3.

| Rank | Peptides Identity within Clusters A and B | Peptides Identity within Clusters C and D | Rank | Peptides Identity within Clusters A and B | Peptides Identity within Clusters C and D |
|---|---|---|---|---|---|
| **1** | KCNA1_438_450 | NOS3_1171_1183 | **41** | VASP_271_283 | TY3H_65_77 |
| **2** | FRAP_2443_2455 | ESR1_160_172 | **42** | PPR1A_28_40 | NFKB1_330_342 |
| **3** | KCNA3_461_473 | ANDR_785_797 | **43** | ESR1_160_172 | NCF1_321_333 |
| **4** | GPR6_349_361 | VASP_271_283 | **44** | NFKB1_330_342 | PTK6_436_448 |
| **5** | RB_242_254 | E1A_ADE05_212_224 | **45** | MPIP1_172_184 | EPB42_241_253 |
| **6** | BAD_69_81 | GBRB2_427_439 | **46** | PLEK_106_118 | ART_025_CXGLRRWSLGGLRRWSL |
| **7** | RB_803_815 | CREB1_126_138 | **47** | ERBB2_679_691 | KCNA3_461_473 |
| **8** | RBL2_655_667 | PTN12_32_44 | **48** | ACM5_494_506 | KCNA2_442_454 |
| **9** | H2B1B_ 27_40 | RYR1_4317_4329 | **49** | NOS3_1171_1183 | FRAP_2443_2455 |
| **10** | ADDB_706_718 | RS6_228_240 | **50** | E1A_ADE05_212_224 | VAPS_150_162 |
| **11** | KS6A1_374_386 | GRIK2_708_720 | **51** | KIF2C_105_118_S106G | PLM_76_88 |
| **12** | CA2D1_494_506 | NCF1_296_308 | **52** | ADRB2_338_350 | REL_260_272 |
| **13** | MBP_222_234 | KCNA6_504_516 | **53** | KAP2_92_104 | GPSM2_394_406 |
| **14** | KAPCG_192_206 | F263_454_466 | **54** | STK6_283_295 | STMN2_90_102 |
| **15** | NR4A1_344_356 | CFTR_761_773 | **55** | RYR1_4317_4329 | DESP_2842_2854 |
| **16** | MARCS_160_172 | KIF2C_105_118_S106G | **56** | NCF1_321_333 | KS6A1_374_386 |
| **17** | MP2K1_287_299 | TOP2A_1463_1475 | **57** | KPB1_1011_1023 | CA2D1_494_506 |
| **18** | ACM4_456_468 | KAP3_107_119 | **58** | CSF1R_701_713 | GYS2_1_13 |
| **19** | ACM1_421_433 | VTNC_390_402 | **59** | CAC1C_1974_1986 | FOXO3_25_37 |
| **20** | H32_3_18 | MYPC3_268_280 | **60** | ART_025_CXGLRRWSLGGLRRWSL | ACM1_421_433 |
| **21** | CGHB_109_121 | MPIP1_172_184 | **61** | VASP_150_162 | ACM4_456_468 |
| **22** | ANXA1_209_221 | K6PL_766_778 | **62** | KAP3_107_119 | BAD_93_105 |
| **23** | KCNA2_442_454 | NMDZ1_890_902 | **63** | EPB42_241_253 | ACM5_494_506 |
| **24** | RAP1B_172_184 | STK6_283_295 | **64** | PTK6_436_448 | ACM5_498_510 |
| **25** | K6PL_766_778 | CSF1R_701_713 | **65** | CFTR_730_742 | MARCS_160_172 |
| **26** | STMN2_90_102 | CGHB_109_121 | **66** | NMDZ1_890_902 | H32_3_18 |
| **27** | PLM_76_88 | RB_242_254 | **67** | PTN12_32_44 | NR4A1_344_356 |

| | | | | | |
|---|---|---|---|---|---|
| **28** | FOXO3_25_37 | RAP1B_172_184 | **68** | CREB1_126_138 | MP2K1_287_299 |
| **29** | ACM5_498_510 | BAD_69_81 | **69** | TY3H_65_77 | RB_803_815 |
| **30** | GYS2_1_13 | GPR6_349_361 | **70** | RS6_228_240 | KAPCG_192_206 |
| **31** | BAD_93_105 | ANXA1_209_221 | **71** | VTNC_390_402 | MBP_222_234 |
| **32** | ANDR_785_797 | LIPS_944_956 | **72** | NCF1_296_308 | PPR1A_28_40 |
| **33** | REL_260_272 | KCNA1_438_450 | **73** | TOP2A_1463_1475 | CDN1A_139_151 |
| **34** | BAD_112_124 | BAD_112_124 | **74** | MYPC3_268_280 | RBL2_655_667 |
| **35** | DESP_2842_2854 | KPB1_1011_1023 | **75** | GRIK2_708_720 | PLEK_106_118 |
| **36** | KPCB_19_31_A25S | CFTR_730_742 | **76** | GBRB2_427_439 | CENPA_1_14 |
| **37** | CENPA_1_14 | SCN7A_898_910 | **77** | KCNA6_504_516 | H2B1B_ 27_40 |
| **38** | LIPS_944_956 | CAC1C_1974_1986 | **78** | CDN1A_139_151 | ERBB2_679_691 |
| **39** | GPSM2_394_406 | ADRB2_338_350 | **79** | F263_454_466 | ADDB_706_718 |
| **40** | SCN7A_898_910 | KAP2_92_104 | **80** | CFTR_761_773 | KPCB_19_31_A25S |

**Table S3.** Differences in peptides phosphorylation ($n = 80$) before and after nivolumab treatment, determined by a Student's paired $t$-test. Peptides are ordered by $p$-value: highest ranking represents lowest $p$-value.

| Rank | Peptide Identity | Rank | Peptide Identity |
|---|---|---|---|
| **1** | VASP_271_283 | **41** | STMN2_90_102 |
| **2** | RBL2_655_667 | **42** | BAD_112_124 |
| **3** | GRIK2_708_720 | **43** | BAD_69_81 |
| **4** | KS6A1_374_386 | **44** | VASP_150_162 |
| **5** | RS6_228_240 | **45** | GBRB2_427_439 |
| **6** | PTK6_436_448 | **46** | GPSM2_394_406 |
| **7** | PLEK_106_118 | **47** | RB_242_254 |
| **8** | NR4A1_344_356 | **48** | MBP_222_234 |
| **9** | KAP2_92_104 | **49** | DESP_2842_2854 |
| **10** | KIF2C_105_118_S106G | **50** | CA2D1_494_506 |
| **11** | ADRB2_338_350 | **51** | CREB1_126_138 |
| **12** | CFTR_761_773 | **52** | NOS3_1171_1183 |
| **13** | CAC1C_1974_1986 | **53** | ACM4_456_468 |
| **14** | NMDZ1_890_902 | **54** | CSF1R_701_713 |
| **15** | TOP2A_1463_1475 | **55** | KCNA2_442_454 |
| **16** | F263_454_466 | **56** | CDN1A_139_151 |
| **17** | RYR1_4317_4329 | **57** | FRAP_2443_2455 |
| **18** | MARCS_160_172 | **58** | E1A_ADE05_212_224 |
| **19** | KAP3_107_119 | **59** | ACM1_421_433 |
| **20** | K6PL_766_778 | **60** | ERBB2_679_691 |
| **21** | KPB1_1011_1023 | **61** | EPB42_241_253 |
| **22** | SCN7A_898_910 | **62** | KCNA3_461_473 |
| **23** | CFTR_730_742 | **63** | PPR1A_28_40 |
| **24** | NCF1_296_308 | **64** | ESR1_160_172 |
| **25** | VTNC_390_402 | **65** | PLM_76_88 |
| **26** | MYPC3_268_280 | **66** | GYS2_1_13 |
| **27** | PTN12_32_44 | **67** | KAPCG_192_206 |
| **28** | NCF1_321_333 | **68** | H2B1B_27_40 |
| **29** | CGHB_109_121 | **69** | RAP1B_172_184 |
| **30** | TY3H_65_77 | **70** | H32_3_18 |
| **31** | LIPS_944_956 | **71** | KPCB_19_31_A25S |
| **32** | STK6_283_295 | **72** | CENPA_1_14 |
| **33** | KCNA6_504_516 | **73** | ACM5_494_506 |
| **34** | NFKB1_330_342 | **74** | GPR6_349_361 |
| **35** | REL_260_272 | **75** | ADDB_706_718 |
| **36** | MPIP1_172_184 | **76** | ACM5_498_510 |
| **37** | KCNA1_438_450 | **77** | MP2K1_287_299 |
| **38** | RB_803_815 | **78** | FOXO3_25_37 |
| **39** | ART_025_CXGLRRWSLGGLRRWSL | **79** | BAD_93_105 |
| **40** | ANDR_785_797 | **80** | ANXA1_209_221 |

© 2019 by the authors. Licensee MDPI, Basel, Switzerland. This article is an open access article distributed under the terms and conditions of the Creative Commons Attribution (CC BY) license (http://creativecommons.org/licenses/by/4.0/).
